# Dual Approach Using Vitrectorhexis Combined with Anterior Vitrectomy in Pediatric Cataract Surgery

**DOI:** 10.1155/2013/124754

**Published:** 2013-02-12

**Authors:** Özgür İlhan, Mesut Coşkun, Uğurcan Keskin, Emre Ayıntap, Nilüfer İlhan, Esra Tuzcu, Mutlu Dağlıoğlu, Hüseyin Öksüz

**Affiliations:** Department of Ophthalmology, Faculty of Medicine, Mustafa Kemal University, Hatay, 31100 Antakya, Turkey

## Abstract

*Purpose*. To evaluate efficacy and safety of vitrectorhexis method for both anterior and posterior capsules combined with anterior vitrectomy in children with cataract. *Methods*. A retrospective chart review was performed for 19 children with cataract operated at a tertiary referral center. Dual approach including anterior and posterior segments was used during the surgery in terms of capsulotomy, intraocular lens (IOL) implantation, and anterior vitrectomy. *Results*. A total of 23 eyes of 19 patients were enrolled in the study. The mean age of the children was 39.4 ± 2.2 months (5–78). The mean postoperative followup duration was 20.6 ± 7.8 months (3–32). Intraoperative tear was observed only in one of 23 (4.3%) eyes during anterior capsulotomy. All of the patients had a clear visual axis and showed no IOL decentration. *Conclusions*. Dual approach using vitrectorhexis and anterior vitrectomy is an easy-to-perform technique that seems safe and effective in the short term for younger children.

## 1. Introduction

Cataract is the most frequent reason for blindness in children and associated with approximately 5%–20% of blindness in childhood in the world [1–3]. It has been reported that 200,000 children are blind due to bilateral cataract worldwide [1] despite the many technical improvements that have occurred in the recent years [4].

There are some important differences between pediatric and adult cataract surgeries. The most noticeable one is anterior capsulotomy step of the cataract surgery. This step is quite significant because long-term centralization and stabilization of a capsular bag fixated intraocular lens (IOL) are associated with the factors including anterior capsulotomy size and shape and integrity of the capsule edge. The response of the pediatric anterior capsule to surgical maneuvers differs from adult. Because of the highly flexible nature of the young capsule, performing the maneuvers of anterior capsule in adult cataract surgery to a pediatric capsule may result in complications [5, 6].

The term “vitrectorhexis” describes a technique that needs vitrector hand piece and has been used to perform anterior capsulotomy for years in pediatric cataract surgery and named formerly as “mechanized anterior capsulotomy” [6, 7]. Vitrectorhexis is a useful alternative to the manual continuous curvilinear capsulorhexis (CCC) for children below 6 years of age when the anterior capsule is very elastic and not easy to control. In children over 6 years of age, the manual CCC is the best technique because control of anterior capsule and completion of capsulotomy are easier [8]. 

There are a limited number of reports investigating the use of vitrectorhexis for both anterior and posterior capsulotomies and anterior vitrectomy in pediatric cataract and IOL implantation surgery. Therefore, the aim of the current study is to evaluate efficacy and safety of vitrectorhexis method for both anterior and posterior capsules combined with anterior vitrectomy in children with cataract.

## 2. Materials and Methods

A retrospective chart review was performed for 19 children with pediatric cataract operated by one surgeon (H. Öksüz) at tertiary referral center between March 1, 2009, and December 31, 2011. Informed consent was obtained from parents of the patients before the surgery. A comprehensive medical history was obtained from parents and an ophthalmological examination was performed on each patient prior to the cataract surgery. Patients who had uveitis, microphthalmos, glaucoma, and corneal disease that prevents adequate visualization of anterior segment of the eye were excluded from the study. Preoperatively, patients had an ophthalmologic examination consisting of a slit-lamp examination, intraocular pressure measurement by Goldmann applanation tonometry, and dilated fundus examination. Mydriatic eye drops (tropicamide 0.5% and phenylephrine hydrochloride 2.5%) were given every 15 minutes for 3 times beginning 2 hours before surgery. Cataract surgeries were performed under general anesthesia, and the same surgical approach was used in each case. The method used in this study is a modification of the technique which was described by Wilson Jr. [6].

Surgical steps were defined as follows. When creating a vitrectorhexis, a vitrector hand piece supported by a venturi pump was used. Two side ports were created at superior-temporal and superior-nasal quadrants by using a 20-gauge side port knife. One of them was used for separate infusion, and the other was used for a vitrector hand piece which matched the gauge of infusion cannula. Hence, ensuring the incisions into the eye provide for a tight fit when the surgical instruments in the anterior chamber. The anterior camber of children narrows rapidly when leakage appears around the surgical instruments, and that makes vitrectorhexis quite difficult to finish. The cutting port of 20-gauge vitrector hand piece placed posteriorly in contact with the surface of the anterior capsule and the cutter turned on and vacuum increased until the anterior capsule was caught and opened. Anterior capsular opening was created by the vitrector, not using cysotome. We used a low cutting rate (200–250 cuts per minute) and high infusion rate (the bottle at the highest level). The level of aspiration maximum varied in between 100 and 250. Later, the capsular opening was enlarged towards periphery until the preferred shape and diameter are achieved. The vitrectorhexis size was left a bit smaller than the diameter of IOL which was used at the surgery. After the hydrodissection, cataractous lens removal and lens cortex aspiration were performed by using bimanual irrigation and aspiration (I/A) cannula. Sodium hyaluronate (1.8%) was injected into the anterior chamber as an ophthalmic viscosurgical device (OVD), and a 2.75 mm corneal incision was created at 12 o'clock quadrant, then a foldable, acrylic, posterior chamber IOL was inserted through the incision, which was closed with 10–0 nylon suture. A sclerostomy was created at pars plicata or pars plana after conjunctival incision. Then, superior-nasal side port was used for separate infusion and the vitrector hand piece introduced into anterior vitreous via sclerostomy. Anterior vitrectomy was performed (500 cuts per minute and 40–50 cm as the bottle height for lower infusion), then posterior vitrectorhexis was performed (200–250 cuts per minute and 40–50 cm as the bottle height for lower infusion) by using the vitrector hand piece. The posterior capsule was compressed in between IOL and the vitrector hand piece. The cutter turned on with the cutting port facing up, and vacuum increased until the posterior capsule was caught and opened. The capsular opening was enlarged towards periphery, and its size was a bit smaller than the anterior vitrectorhexis. The sclerostomy and conjunctiva were closed by 7/0 vicryl suture. Sodium hyaluronate (1.8%) was removed by using I/A, and stromal hydration was performed with balanced salt solution. Whether the side port had a leakage, it was sutured by 10–0 nylon suture. Lomefloxacin and prednisolone acetate were given 8 times per day.

## 3. Results

All cataractous eyes were congenital and developmental cases diagnosed between 5 and 78 months of age. A total of 23 eyes of 19 patients (8 girls and 11 boys) were enrolled in the study. Fifteen of the patients had unilateral involvement, and the remaining 4 patients had bilateral involvement. Overall, the mean age of the children was 39.4  ±  2.2 months (5–78 months). The mean postoperative followup duration of the patients was 20.6  ±  7.8 months (3–32 months).

**Figure 1:**
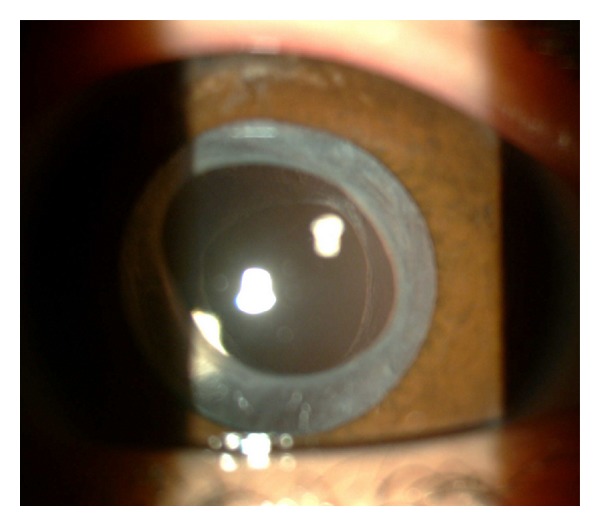
A slit-lamp photograph showing clear visual axis, ACCC, and PCCC in a 60-month-old child at 2 years followup. (ACCC: anterior continuous curvilinear capsulorhexis PCCC: posterior continuous curvilinear capsulorhexis).

Intraoperative tear was observed only in one of 23 (4.3%) eyes during anterior capsulotomy. No other complication was observed intraoperatively. Mild anterior chamber reaction was seen in 5 patients operated before 36 months of age at early postoperative period. There was no major postoperative complication including pupillary synechia, capture, distortion, and PCO during the followup. All of the patients had a clear visual axis and showed no IOL decentration throughout the followup period (Figure 1).

## 4. Discussion

Performing a continuous curvilinear capsulorhexis in children eyes is very difficult. However, alternative methods for opening the anterior capsule of pediatric cataracts have been researched by eye surgeons. Vitrectorhexis, Fugo plasma blade, and radio-frequency diathermy are alternative methods and can be used presently. These alternative methods, CCC, and can-opener technique are investigated and compared experimentally in a porcine model by Wilson Jr. [6]. The architecture of the capsular edge and extensibility of the capsule were evaluated for each method. The CCC is of more regular edge surface and more extensible than the others [5, 6]. Moreover, the CCC is gold standard technique and has been used by many surgeons for pediatric cataract surgery in children after 2 years of age [4, 6]. On the other hand, it has been showed that vitrectorhexis technique is frequently used for anterior capsulectomy in pediatric cataracts due to the ease of performance in children younger than 2 years of age [6].

Although there were some studies which reported capsulectomy techniques using the vitrector hand piece in pediatric cataract surgery (no IOL implantation) published at the beginning of the 1980s [9, 10], mechanized anterior capsulotomy combined with IOL implantation in pediatric cataract surgery was first reported in the 1990s [7, 11].

Wilson Jr. [6] compared mechanized anterior capsulectomy and manual CCC in a laboratory setting in 2004. The vitrector handpiece was begun to be used for performing mechanized anterior capsulectomy due to difficulty of manual CCC on the younger children having highly elastic capsule. Eighteen pairs of postmortem children eyes (range: 4 days−16 years) underwent pediatric cataract surgery (with IOL implantation) within a day of enucleation. One eye of each pair had a mechanized anterior capsulotomy, and the other eye had a CCC. The anterior capsular integrity was evaluated at each step of the surgery in terms of radial tears. The development of radial tear was observed in one of 18 children eyes with mechanized anterior capsulectomy, whereas it was not observed in eyes with manual CCC. The tear was occurred in a 16-year-aged eye. However, in 6 eyes, the manual CCC was not continued in a round manner and reached out the lens equator and all these 6 wrong capsulotomies happened in eyes younger than 5 years old. 

Subsequently, a study comparing anterior vitrectorhexis and the manual CCC in pediatric cataract surgery showed that 19 of 339 eyes (5.6%) had an anterior capsule tear (in vitrectorhexis group, 12 of 226 eyes 5.3% and in CCC group, 7 of 113 eyes 6.2%). Anterior capsule tears were observed during anterior capsulotomy in 7 eyes, hydrodissection in 1, cataract removal in 3, and IOL delivery/manipulation in 8. The study suggested that vitrectorhexis is quite appropriate for use in children below 6 years of age because of having very elastic anterior lens capsule. Manual CCC was the best option for 6 years old and older age groups [8]. In the present study, only 1 of 23 (4.3%) eyes was noted to develop an anterior capsule tear. This tear occurred during anterior capsulotomy step of a child aged 78 months old. There was no an anterior capsule tear during remaining steps of surgery (hydrodissection, cataract removal, and IOL delivery/manipulation). Especially, there was no anterior capsule tear during IOL insertion and manipulation step of the surgery, and it was likely associated with routine use of cartridge or injector systems. 

One of the most common and important complications after cataract surgery in children is PCO [9, 12]. The incidence of PCO has been reported from 16% up to 50% at several studies in which anterior continuous curvilinear capsulorhexis (ACCC), PCCC, and anterior vitrectomy were performed (no IOL implantation) [13, 14]. A study reported that approximately 100% PCO rate was found in the patients without performance of an anterior vitrectomy [15]. However, another study reported that PCO rate was 9% when pars plana capsulectomy and anterior vitrectomy were performed [16]. In the current study, PCO development was not observed during the followup period.

The outcome of cataract surgery in children appreciably depends on management of the posterior capsule. PCO occurs quickly and nearly unavoidable in very young children when the posterior capsule is left intact after cataract removal [4, 17]. Despite the presence of some novel noninvasive methods for the prevention of PCO [18], primary posterior capsulotomy and anterior vitrectomy are essential surgical interventions for eyes with pediatric cataract, particularly in younger age group [17]. Management of the posterior capsule is frequently applied during the surgery [4, 9, 19]. When anterior segment approach is used, the posterior capsulotomy is performed before IOL insertion. Posterior capsulotomy and anterior vitrectomy are commonly carried out after implantation of IOL when posterior segment approach is used. A 3.5–4 mm round and central posterior capsulotomy should be targeted. There are various techniques including vitrectorhexis, posterior continuous curvilinear capsulorhexis, Fugo plasma blade, and radio-frequency diathermy for posterior capsulotomy as their usage for anterior capsulotomy [20]. In the current study, vitrectorhexis was performed for posterior capsulotomy because it was easier to perform than the manual CCC according to the study by Wilson Jr. [6]. 

Even though many studies [21–23] showed that PCCC is an effective technique to prevent PCO development in children with pediatric cataract by avoiding lens epithelial cells from proliferating over the visual axis, secondary cataract might still occur because lens epithelial cells can regenerate, grow, and migrate on top of the anterior hyaloid face which likely needs an anterior vitrectomy, principally in younger children [21, 24, 25]. Additionally, the inflammatory reaction in younger children is frequently severe, and cyclitic membranes might occur on top of the anterior vitreous surface that contribute to PCO development. Therefore, an anterior vitrectomy application should be performed in younger children [20]. In the present study, all of the patients had anterior vitrectomy procedure that was performed via pars plana and plicata. Pars plicata approach was only used in infancy because pars plana vitrectomy is applicable when the patient is at least a 6-month-old full-term infant [26]. A detailed fundus examination was performed to the patients having pars plicata vitrectomy due to likelihood of retinal break at followup visits.

In another study, 16 eyes of 10 patients with a mean age of 4.3 years underwent a lensectomy and an anterior vitrectomy procedure through the pars plana approach, followed by implantation of a posterior chamber IOL to the sulcus over the double capsules [27]. All the patients had a 6.5 mm polymethyl methacrylate posterior chamber IOL. There was no secondary cataract formation obscuring the visual axis during the mean followup period of 79.2 months. Although the study suggested that pars plana lensectomy with double-capsule-supported intraocular lens implantation method is an easy and safe way in children, this technique has several disadvantages. One of them is the implantation of IOL to the sulcus which may cause chronic inflammation due to the contact of neighboring ocular tissues with the IOL. Another disadvantage is residual lens cortex located peripherally which also may lead chronic inflammation. Additionally, IOL dislocation may occur due to the absence of fibrosis in the sulcus region [27].

To the best of our knowledge, this is the first report investigating vitrectorhexis for both anterior and posterior capsulotomies and anterior vitrectomy in pediatric cataract surgery. The technique used in the current study has several advantages. First, the use of infusion for vitrectorhexis and usage of small amount of OVD only in IOL implantation may facilitate removal of inflammatory mediators and lead to less postoperative inflammation. Second, an anterior vitrectomy via posterior route after the implantation of IOL prevents the vitreous prolapse into the anterior chamber during the surgery. The likelihood of the development of vitreous strands reaching to the anterior segment or to the corneal wound edges was reduced as low as possible by the technique because of many post-operative complications including cystoid macular edema and retinal detachment associated with these vitreous strands.

Although the present study has several advantages, there are some limitations. First, the number of the patients is not sufficiently high in the study. Thus, future studies with larger groups may be recommended to reveal a more definitive conclusion. The absence of a control group is the second limitation. Thirdly, the study period had a mean of 20.6 months followup, and future studies with a longer period might be preferred. However, most of the pediatric cataract surgeons consider that followup time more than 2 years is recommended to reveal reliable conclusions. We still think that more than one and a half years of followup in the current study might give some valuable conclusions, and absence of PCO development in the patients seems remarkable at least in short term. Fourth, determination of PCO development in the study was made by using only slit-lamp examinations. More sophisticated methods evaluating the development of PCO can give more valuable outcomes. 

In conclusion, dual approach using vitrectorhexis and anterior vitrectomy is an easy-to-perform technique that seems safe in the short term in children less than 6 years of age. The main advantages of this technique are to ease both anterior and posterior capsulotomies and possibly the prevention of the development of PCO in pediatric cataract cases. Prospective controlled studies with a larger number of patients and longer durations of followup are still needed in the future to verify the advantages of this dual technique in younger children with cataract.
